# Formaldehyde Exposure and Its Potential Health Risk in Some Beauty Salons in Kumasi Metropolis

**DOI:** 10.1155/2020/8875167

**Published:** 2020-11-05

**Authors:** Noah Kyame Asare-Donkor, James Kusi Appiah, Vincent Torve, Ray Bright Voegborlo, Anthony Apeke Adimado

**Affiliations:** Department of Chemistry, Kwame Nkrumah University of Science and Technology, Kumasi, Ghana

## Abstract

Cosmetologists may be potentially exposed to high levels of formaldehyde as a result of their exposure to formaldehyde released from the various cosmetic products used in the beauty salons. In order to assess the exposure of cosmetologists to formaldehyde, the indoor air in sixty beauty salons across the ten submetros in Kumasi were sampled to determine the formaldehyde levels and the associated noncarcinogenic human health risks. Sampling was done using System Service Innovation Incorporation air sampler model 1000*i*, and the MBTH spectrophotometric method was used for analysis. The mean levels of formaldehyde concentrations ranged from 88.67 to 170.67 *µ*g/m^3^. Out of the sixty salons sampled, 36 salons had formaldehyde levels above the WHO permissible limit of 100 *µ*g/m^3^ for an eight-hour working period and also exceeded the 55 and 9 *µ*g/m^3^ for chronic and acute reference exposure limit, respectively, set by the Office of Environmental Health Hazard Assessment. The results of this study revealed that the number of customers that visit the salon in a week, number of salon services offered, and age of salon had a positive significant correlation with the level of formaldehyde determined in each salon. The health risk study also revealed that about 50% of the salons had hazard quotient (HQ) above the safety limit (HQ = 1) and may, therefore, pose health risks to cosmetologists in these salons. Results from the analysis of the questionnaire revealed that hairdressers in salons that provide the entire range of salon services captured in the study are at higher risk to the effects of formaldehyde.

## 1. Introduction

Formaldehyde has been classified as a Group one human carcinogen by the International Agency for Research on Cancer [1]. There exist high formaldehyde emitters in the indoor environment with a low level of air exchange rates (ventilation) than in the outdoor environment [2]. Even though rank profiling of chemicals and exposures that are of concern is difficult and unknown, the Scientific Committee on Health and Environmental Risks (SCHER) states that formaldehyde such as carbon monoxide, nitrogen dioxide, benzene, naphthalene, environmental tobacco smoke (ETS), radon, lead, and organophosphate pesticides is a compound of concern in the indoor environment [3].

At low concentrations, formaldehyde is safely and legally used in cosmetics as it is released in small amounts over time to help protect cosmetic products against contamination by bacteria during storage and during continued usage. Aside from free FA, cosmetics are also preserved by several formaldehyde donors known as formaldehyde-releasing preservatives, which slowly release formaldehyde through degradation or decomposition under usage conditions [4]. Formaldehyde donors such as quaternium-15 (QU), imidazolidinyl urea (IU), diazolidinyl urea (DU), dimethyloldimethylhydantoin (DMDM), and 2-bromo-2-nitropropane-1, 3-diol (bronopol, BP) are the most commonly used to prevent the growth of micro-organisms in cosmetic products. The antimicrobial activity of these preservatives probably results from formaldehyde released by hydrolysis in the presence of water. Moreover, the amount of formaldehyde released by these formaldehyde donors may be dependent on various variations such as the nature and concentration of the releaser, pH, temperature, and storage time [5]. In beauty salons, a wide diversity of chemical products is used in the different therapies such as facial cleansing, skin, nails and body hydrotherapy and care, antiwrinkle, pigmentation and acne treatment, makeup, depilation, body and face massage, reflexology, aromatherapy, and face and body hair removal among many others. In Ghana, beauty salons represent one of the significant and growing occupations in the country with the workers being mostly women. In beauty salons, the two main categories of chemical products used that contain high amounts of formaldehyde are hair straightening/smoothening treatments and nail hardeners.

The majority of formaldehyde exposures occur through inhalation, the dermal and eye contact. In the beauty salons, the level of exposure depends upon the products used, stylist techniques, and ventilation. People who become sensitized to formaldehyde may experience headaches and minor eye and airway irritation even at relatively low levels [6]. The irritant effects of formaldehyde are well documented, with reports of eye, nose, and throat irritation, loss of sense of smell, increased upper respiratory disease, dry and sore throats, respiratory tract irritation, cough, chest pain, shortness of breath, and wheezing. Salon workers' complaints such as nosebleeds, eye irritation, and difficulty in breathing after using Brazilian Blowout hair smoothing or straightening product have been reported [7].

Cosmetologists and beauticians and to some extent customers are exposed to high concentrations of several compounds that are included in the various chemical products used in their work or treatments. Each of the products has a large number of components including several volatile organic compounds (VOCs), including formaldehyde. The variations of chemical exposures have been described in a few studies mainly focused on hairdressing and nail salons [8]. Coppola, Global Keratin, La Brasiliana, and Brazilian Blowout are all hair-straitening products that have been found to contain formaldehyde with Global Keratin and Brazilian Blowout containing levels that exceed OSHA limit of 0.5 ppm which calls for “potential cancer” to be included on the product label [7].

Working as a hairdresser or a beautician has been associated with adverse reproductive health effects such as spontaneous abortion, congenital malformations, childhood cancer, and a developmental problem [6].

Formaldehyde is electrophilic and can react with biogenic compounds in the body which are nucleophilic. Small amounts of formaldehyde are produced from methanol via the enzyme alcohol dehydrogenase (ADH), which is a human metabolite and can be measured in urine [3]. Endogenously, formaldehyde produced in humans is an essential intermediate in the production of purines, thymidine, and some amino acids and this is produced in all metabolically active cells with levels in blood ranging from 2–3 mg/L. Formaldehyde reacts rapidly with primary and secondary amines, thiols, hydroxyls, and amides, forming methylol derivatives at the site of contact. It may also react with DNA, RNA, and protein to form adducts or cross-links [9].

According to a report by “Health Canada” based on human clinical studies and on animal experiments, the primary effects of acute exposure to formaldehyde are the irritation of the mucosa of the upper respiratory tract and the eyes. In both humans and experimental animals, formaldehyde is absorbed easily by all exposure routes, thus inhalation, and dermal and oral routes. Formaldehyde has a high solubility in water, and this causes rapid absorption of it in the respiratory and gastrointestinal tract where it can be oxidized to form formate and exhaled as carbon dioxide or incorporated in biological matrices. Once formaldehyde is inhaled, it quickly reacts at the site of contact and is readily metabolised in the respiratory tissue. As a result of formaldehyde reactivity in target tissues, direct contact with it causes local irritation, and acute and chronic toxicity and has genotoxic and cytotoxic properties [10]. The major concerns of repeated and chronic exposure to formaldehyde are sensitization and cancer. In sensitized persons, formaldehyde can cause asthma and contact dermatitis. In persons who are not sensitized, prolonged inhalation of formaldehyde at low levels is unlikely to result in chronic pulmonary injury. Adverse effects on the central nervous system, such as increased prevalence of headache, depression, mood changes, insomnia, irritability, attention deficit, and impairment of dexterity, memory, and equilibrium have been reported to result from long-term exposure [11]. Based on available nasopharyngeal cancer data, formaldehyde is regarded as a possible human carcinogen following inhalation exposure and as such is classified in Group one of human possible carcinogen by the International Agency for Research on Cancer (IARC).

However, in Ghana, no such guideline value has been established and this necessitates the need to monitor formaldehyde levels indoor as one-step research towards that. The objective of this study is to assess the formaldehyde exposure and its potential health risk in some beauty salons in Kumasi metropolis.

## 2. Materials and Methods

### 2.1. Study Area and Sampling Sites

The Ashanti region of Ghana has a projected total population of 5,406,209 [12]. Kumasi is the capital city of Ashanti region and is the second-largest city in the country. Kumasi is approximately 300 miles (480 km) north of the equator and 100 miles (160 km) north of the Gulf of Guinea. It is popularly known as “The Garden City” or “Heart Beat” of Ghana because of its many beautiful species of flowers and plants [13].

### 2.2. Sampling and Sample Preparation

Air samples were taken from sixty salons located in the ten submetros that make up the Kumasi metropolis. Within each submetro, samples were obtained from six salons scattered throughout the submetro. The various services offered by these salons include hair washing, retouching, styling, braiding, dying/colouring, smoothening/straightening, pedicure, manicure, and makeup. Majority of the salons where sampling was done were located in container shops sited along roadsides. Samples were taken from the centre area of the salon. A System Service Innovation Incorporation air sampler model 1000*i* was used for sampling. Thirty-five millilitres (35 ml) of MBTH solution was placed in an impinger of the sampler. The air inlet of the air sampler was placed at a height of 1.5 meters above the ground level and approximately in the centre of each salon. Air was drawn through the MBTH solution at a rate of 0.5 L/min for 30 mins. After 30 minutes of sampling, the absorbing MBTH solution was transferred into an air-tight sample container which was then taken to the laboratory for analysis. During the 30-minute sampling period, a 433 MHz electronic weather station was used to record the temperature and humidity of the sampling facility. Before sampling, fans were switched off and doors and windows were also closed.

The levels of formaldehyde were in the volume of air was obtained through the following calculations.

Conversion of volume of air sampled to the volume of air at standard conditions is as follows:

(1) $$\begin{array}{c} V_{s}=\frac{P\times V\times 298}{101\left(T+273\right)}, \end{array}$$

where *V*_*s*_ = volume of air at standard conditions (101 kPa and 298 K) (*L*); *V* = volume of air sampled (*L*); *P* = barometric pressure, kPa; and *T* = temperature of sample air, °C.

Calculation of total micrograms of formaldehyde collected in each impinger sample is as follows:

(2) $$\begin{array}{c} C_{t}=C_{a}\times F_{a}, \end{array}$$

where *C*_*t*_ = total formaldehyde in the sample (*μ*g) and *C*_*a*_ = total quantity of formaldehyde in the sample aliquots taken from the impinger as determined from the calibration curve as follows:

(3) $$\begin{array}{c} C_{a}=\frac{\text{absorbance}-0.009}{0.2014}\;\mu g.\\ F_{a}\text{ is aliquot factor}=\frac{\text{sampling solution volume},\text{ mL}}{\text{aliquot used},\text{ mL}}. \end{array}$$

Calculation of the formaldehyde concentration in the volume of air sampled at the salon is as follows:

(4) $$\begin{array}{c} C_{L}=\frac{C_{t}\times 24.47}{V_{s}\times 30.03}, \end{array}$$

where *C*_*L*_ = parts of formaldehyde per million parts air (ppm); 30.03 = molecular weight of formaldehyde; 24.47 = *μ*L of formaldehyde gas in 1 *μ*mol at 101 kPa and 298 K; and *C*_*L*_ is converted to mg/m^3^ by way multiplying by 1.23, and mg/m^3^ is converted to *µ*g/m^3^ by dividing by 1000.

### 2.3. Quality Control and Assurance

A series of quality assurance and control processes were followed both in the laboratory analysis of samples and on the field during sampling to ensure that results obtained from the study were accurate and precise. All samples were quantified with multipoint calibration curves from pure chemicals of analytical grade. Analytical blanks were included in all analysis. Background correction of the spectrophotometer was also performed before any analysis was done.

### 2.4. Administration of Questionnaire

The questionnaire was administered at each sampling facility visited to gather information about the workers, working practices, and the facility. Information such as the age of the salon, services offered, various cosmetic products used in the salon, number of years of working experience, knowledge about the products used, and health conditions experienced during the period of work among others was gathered.

### 2.5. Health Risk Assessments

#### 2.5.1. Potential Dose (pd)

Potential dose is the determination of potential exposure, which indicates an effective dose, in biological terms, of a pollutant that could cause human health effects in a given environment. Mathematically, exposure (pd) for an individual (*i*) because of the admissions process (inhalation or ingestion) can be calculated [14, 15]:

(5) $$\begin{array}{c} PD_{i}=C_{j}\times {\text{IR}}_{i}\;\times \;T_{ij}, \end{array}$$

where *C*_*j*_ is the concentration of pollutants (*μ*g m^−3^), IR is the rate of contact (m^3^ h^−1^), and *T* is the exposure time (h day^−1^). Due to the difficulty of accuracy measuring the correct rate of inhalation for each individual, the PD was estimated for an exposure period of 8 h, and an IR of 1.02 m^3^ h^−1^ (average inhalation) was used as suggested by the exposure handbook factors [16, 17].

#### 2.5.2. Evaluation of Health Risk

The calculation of the exposure for the lifetime of formaldehyde was estimated by chronic daily intake (CDI) in accordance with the following equation [18]:

(6) $$\begin{array}{c} \text{CDI}=\frac{\left(\text{CA }\times \text{ IR }\times \text{ ED}\times \text{ EF }\times \;L\right)}{\left(\text{BW}\times \text{ ATL }\times \text{ NY}\right)}, \end{array}$$

where CA = chemical concentration in air (mg/m^3^) at sampling site; IR = inhalation rate (m^3^/hr) = 1.02 m^3^/hr; ET = exposure time (hours/days) = 8 hours; EF = exposure frequency (days/yrs) = 365 days/year; ED = exposure duration (years) = 70 years; BW = body weight (kg) = 70 kg; ATL = averaging time (period over which exposure is averaged days); *L* = length of exposure = 40 year; and NY = number of days per year = 365 days year^−1^.

Cancer risk (CR) was then estimated by CDI multiplied by the slope factor (SF) according to the Integrated Risk Information System (IRIS) [19, 20]:

(7) $$\begin{array}{c} \text{CR}=\text{CDI}\times \text{SF}. \end{array}$$

According to the IRIS system, the slope factors in this study for formaldehyde are 0.0455 mgKg^−1^day (US EPA [15–20]).

## 3. Results and Discussion

**Figure 1:**
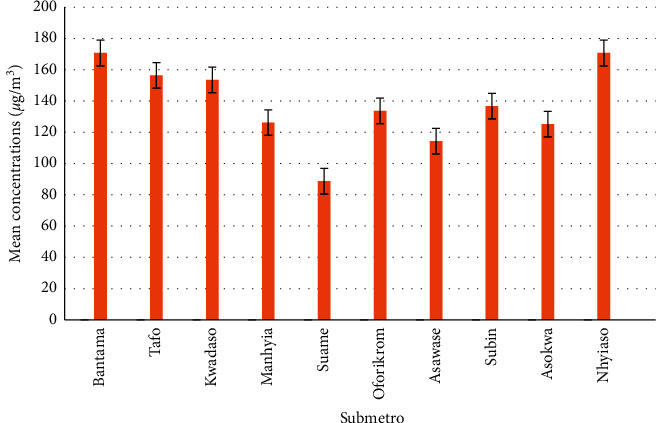
Graph of mean concentrations in the various submetros.

The results for the levels of formaldehyde in the various salons within the various submetros are given in Table 1 and Figure 1. A study by characterizing chemical exposures in hairdressing salons revealed that increase in the number of chemical services offered in a day results in a higher level of chemical exposure. In the salon, the number of services offered in a day is directly linked to the number of customers visiting [6]. The concentrations of formaldehyde determined from the salons at Bantama submetro ranged from 33 to 434 *µ*g/m^3^ with a standard deviation of 152.42 *µ*g/m^3^. This large deviation is indicative of the fact that more cosmetics are being used in some of the salons than the others which is also linked with the number services offered at the individual salons and the number of customers patronizing those salons. The level of formaldehyde concentration determined at the six salons at Tafo submetro ranged from 105 to 200 *µ*g/m^3^ with a standard deviation of 42.43 *µ*g/m^3^. This small deviation may be indicative of the fact that similar quantities of cosmetics are being used in these salons. All the salons sampled in this area offered the similar services hence the difference in the formaldehyde levels determined could, therefore, be attributed to the number of customers that visit each salon, age of facility (salon), and the level of expertise of the workers as indicated by years of working experience. Although ventilation systems in place in each of the salons could be a key factor in the level of formaldehyde determined that may not be the case at all the salons sampled use the same ventilation system (windows and ceiling fan) as observed during sampling.

The levels of formaldehyde concentration from the six salons sampled at Kwadaso, Manhyia, Suame, Oforikrom, Asawase, and Subin submetros ranged from 24 to 349, 102 to 166, 21 to 166, 81 to 327, 21 to 268, and 24 to 265 *µ*g/m^3^, respectively with deviations ranging from 23.47 to 110.84 *µ*g/m^3^. These observations might be attributable to subtle to significant differences in the services being offered, numbers of visits by customers, age of facility, and expertise of the worker as indicated by the number of years of working experience.

The levels of formaldehyde concentration determined from the salons sampled at Asokwa submetro ranged from 97 to 157 *µ*g/m^3^ with a standard deviation of 25.62 *µ*g/m^3^. This subtle deviation observed may be attributed to the similarity and quality in the services provided these salons. All the salons sampled in this area offered two or more services with two of them being the most visited and hence having relatively higher levels of formaldehyde concentrations.

The levels of formaldehyde concentration determined from the six salons sampled at Nhyiaso submetro ranged from 35 to 187 *µ*g/m^3^ with a standard deviation of 152.41 *µ*g/m^3^. This large deviation may again be attributed to the fact that more cosmetics are being used in some of the salons than the others. This can be explained by the number services offered at the individual salons and the number of customers patronizing those salons. Most of the salons sampled in this submetro offered two or more services including hair, nail, and makeups. Acute exposure to formaldehyde concentration of greater than 0.1 mg/m^3^(0.08 ppm) can lead to irritation or contact allergy on various parts of the body while certain types of cancers, asthma, and reproductive and developmental toxicity may result from sustainable exposure [4]. Consequently, several safety and occupational health authorities worldwide have established permissible exposure levels of formaldehyde by inhalation. Such occupational threshold limit values (TLVs) are often categorized as a time-weighted average (TWA), short-term exposure limit (STEL), and ceiling (C) values, with the last defining the exposure limit, which should not be exceeded at any time [3].

For instance, the Occupational Safety and Health Administration (OSHA) has set the STEL for formaldehyde at 2 ppm in 15 min and the permissible exposure limit time-weighted average (PEL-TWA) at 0.75 ppm. The TLV-C proposed by the American Conference of Governmental Industrial Hygienists (ACGIH) is 0.3 ppm. The National Institute for Occupational Safety and Health (NIOSH) has set a more stringent STEL of 0.1 ppm and a recommended exposure limit for occupational exposure of 0.016 ppm. In countries such as the U.K., the Committee on the Medical Effects of Air Pollutants (COMEAP) recommended a limit value of 100 *μ*gm^−3^ (0.5 h) for indoor formaldehyde in 2004. The Ministry of Health and Welfare (MHW) of Japan had established an indoor air guideline value of 0.08 ppm (0.5 h) in June 1997 [3]. In Korea, the indoor formaldehyde was set at 0.1 ppm (8 h) by the Air Quality Standard in Office and Indoor Air Quality Management Act in 2004.

The general public may be exposed to exogenous formaldehyde from contact with consumer products containing formaldehyde or from a range of indoor air sources such as cosmetics used in beauty salons that contain formaldehyde. Indoor air has been estimated to contribute to 98% of total inhalation exposure to formaldehyde. Levels of formaldehyde in ambient air are generally below 0.01 mg/m^3^ but may reach 0.02 mg/m^3^ in urban or industrial areas [10].

### 3.1. Noncarcinogenic Human Health Risk Assessment

The formaldehyde concentrations were used to assess human health risk through inhalation. The characterization of noncarcinogenic health risks for humans consisted of calculations of hazard quotient (HQ), which is defined as the relation between the predicted exposure concentration and the inhalation reference dose (RfDinh). The RfDinh value of 9.83 *μ*g/m^3^ used to calculate HQ was taken from the US EPA [21].  Table S1 shows the HQ values calculated for the formaldehyde concentrations determined for each salon in the study. The HQ values calculated ranged from 0.2 to 4.2 with a mean value of 1.3. About 50% of the calculated HQ values were above the safety limit (HQ = 1). This means that the formaldehyde levels determined in half of the salons sampled in this study may pose a health risk to workers.

### 3.2. Responses Provided by the Respondents to the Questionnaire Administered

Table 2 shows the summary and statistical analysis of the responses provided by the respondents to the questionnaire administered.

The data depict that all the salons offer hair services, and this represents 47% of the total number of services offered by the salons. Nail services represent 35.7% followed by makeups (11.6%), eyelash/eyebrow (5.4%), and skin (1%) which is offered by only one salon. The average number of people (customers) that come to the salon in a week as revealed by the questionnaire administered depicts that the average majority (46%) of the respondents (salon owners/workers) revealed that 20 to 29 of customers visit their salons in a week with only one (1.7%) of the respondents asserting that 50 and more customers visit her salon in a week. Salon owners/workers further asserted that most visits by the customers to their salon are on weekends which start from Friday evening to Sunday evening during which most events such as weddings, funerals, and parties among many others are held.

The working experience among workers of the salons as obtained from the questionnaire shows that majority of the beauty salons (exactly 47) to be precise representing 78.3% are below 5 years with the remaining 13 salons with a percentage of 21.7% being more than 5 years. Most of the respondents had worked between 0 and 5 years with only one (1) working for 16 years and above in the salon environment. The educational background of the hairdressers was ascertained from the questionnaire administered suggested that majority of salon workers (83.3%) completed JHS, SHS (5%), and NVTI (10%) while none (1.7%) had no formal education at all.

98% of the hairdressers in the salons sampled do not have knowledge about the chemical composition of the products used with only one representing 2% having knowledge about the chemical composition of the products used.

The various health effects experienced by the salon workers during their line of work as ascertained with the help of the questionnaire administered indicated that the experienced health effects in order of magnitude are respiratory, eye, nose, and skin irritations with headache being the least. Nausea, vomiting, and asthma were reportedly not experienced by any of the workers as shown in the table. Most of the health effects experienced in the salons may be associated with the inhalation and dermal contact of formaldehyde.

It is observed that the formaldehyde levels determined in *µ*g/m^3^ from the sixty salons ranged from 21.00 to 434.00 with a median of 108.00 and an average value (mean) of 130.53 with a corresponding average temperature of 32.5. Similarly, the standard deviation is an expression that reflects the degree to which the different (observed) values of the variable vary from the average, and the standard deviation value of 81.10 determined for the formaldehyde concentration levels measured implies that the data set is spread apart.

### 3.3. Correlation between Dependent Variables and Independent Variables

The correlation between formaldehyde levels and variables of the study was carried out with SPSS version 17, and the results are presented in Table 3.

It can be observed from Table 3 that there is a positive correlation between the level of formaldehyde concentration and all the independent variables. Since all the Pearson correlation coefficients are positive, it implies that as the dependent variable increases in value, formaldehyde concentration also increases in value. Similarly, as the dependent variables decrease in value, the level of formaldehyde concentration also decreases in value. For instance, the average number of customers in a week showed a significant positive correlation of 0.000 (*p* < 0.05) with the level of formaldehyde concentration.

Moreover, the Pearson correlation coefficient for formaldehyde and an average number of customers in a week is 0.545 which is close to 1; therefore, there is a strong level of significance for the positive correlation between formaldehyde concentration and average number of customers in a week.

The Pearson correlation coefficient for formaldehyde and academic qualification is 0.087 which is close to 0; therefore, there is a weak level of significance for the positive correlation between the level of formaldehyde concentration and academic qualification.

The positive statistically significant correlation between formaldehyde levels and the average number of customers in a week could be due to the fact that the more customers you have in a particular salon, the more cosmetic products that are going to be used which could, in turn, result in an increased level of formaldehyde released. Moreover, more customers in a particular salon could also mean that different brands of cosmetic products are being used since each customer has his/her preferred suitable brand of cosmetic product and each might have a different level of formaldehyde as a preservative. Therefore, the usage of different brands of cosmetics could also result in an increase in the level of formaldehyde released.

### 3.4. Data Analysis and Modelling

#### 3.4.1. Statistical Identification of the Model

The multiple linear regression model which is nonparametric and widely used in research to determine what independent variables have an influence on dependent variables was employed in the study. The formula of the multiple linear regression model is given as

(8) $$\begin{array}{c} y=\beta_{0}+\beta_{1}x_{1}+\beta_{2}x_{2}+\cdots +\beta_{k}x_{k}+\varepsilon . \end{array}$$

This selects among the covariates, those that have a higher influence on the level of formaldehyde concentration in the beauty salons. The model, analysis of the covariates, *X*, is used to determine their significance in determining the level of formaldehyde concentration (CH_2_O) which is our dependent variable:

(9) $$\begin{array}{c} CH_{2}O\;=\;\beta_{\text{0}}+\beta_{1}X_{1}+\beta_{2}X_{2}+\beta_{3}X_{3}+\beta_{4}X_{4}+\beta_{5}X_{5}+\beta_{6}X_{6}+\varepsilon , \end{array}$$

where *X*_1_ is temperature (°C); *X*_2_ is academic qualification; *X*_3_ is years of work experience; *X*_4_ is age of facility; *X*_5_ is no. of service; *X*_6_ is average no. of customers in a week, *ε* is an error term; *β* is regression coefficient; and *β*_0_ is the predicted value of formaldehyde.

#### 3.4.2. Parameter Estimation and Model Selection

These were done using computer software package SPSS Version 17. The variables used in the analysis were the average number of customers in a week, age of facility (beauty salon), and number of services are useful to predict the level of formaldehyde concentration in beauty salons. The backward criteria method was used in the selection process. Variable whose probability value was greater than an alpha value of 10% (0.10) was excluded, and those whose probability values are less than an alpha value of 5% (0.05) were included in the multiple linear regression model.  Table 4 shows the multiple linear regression model summary and overall fit statistics. It was found out that the adjusted *R* of the model 1 is 0.690 with the *R*^2^ = 0.417. This means that the linear regression explains 41.7% of the variance in the data.

The linear regression's *F*-test (Table 5) has the null hypothesis that the model explains zero variance in the dependent variable (in other words, *R*^2^ = 0). The *F*-test is highly significant; thus, we can assume that the model explains a significant amount of the variance in the level of formaldehyde concentration with the independent variables.

Table 6 shows the multiple linear regression estimates including the intercept and the significance levels.

In the multiple linear regression analysis, a nonsignificant intercept was observed, but age of facility coefficient was highly significant, which we can interpret as for every 1-unit increase in age of a beauty salon, we will observe 62.855 additional level of formaldehyde concentration.

Comparison of all variables in the multiple linear regression shows that only age of facility, number of services, and average number of customers in a week are significant predictors. It was also observed that an average number of customers in a week has a higher impact than age of facility and number of services by comparing the coefficients (beta = 0.436 versus beta = 0.322 and 0.214).

Table 7 shows the number of variables that were removed from the model based on their significant level.

It was observed that the significant values for years of working experience, academic qualification, and temperature (°C) are all greater than the alpha value of 5% (0.05) indicating they are not significant.

Also, comparison of the significant values of all the three independent variables shows that years of working experience has the highest significant value of 0.912 as compared to 0.259 and 0.144. It was therefore removed first followed by academic qualification and temperature (°C). The information in Table 7 also allows us to check for multicollinearity in our multiple linear regression model. Tolerance should be  > 0.1 for all variables.

## 4. Conclusions

The mean levels of formaldehyde concentration determined in the sixty salons studied in the ten submetros in the Kumasi Metropolis ranged from 88.67 to 170.67 *µ*g/m^3^. Out of the sixty salons sampled, 36 salons had formaldehyde levels above the WHO permissible limit of 100 *µ*g/m^3^ for an eight-hour working period and also exceeded the 55 *µ*g/m^3^ and 9 *µ*g/m^3^ chronic and acute reference exposure limit set by the Office of Environmental Health Hazard Assessment for prevention of health risk to humans. Only 24 salons had formaldehyde levels below the WHO permissible limit of 100 *µ*g/m^3^ for the eight-hour working period. The number of customers that visit the salon in a week, a number of salon services offered, and age of salon had a positive significant correlation with the formaldehyde levels determined. The health risk study conducted using HQ revealed that the formaldehyde levels pose a health risk to workers in about 50% of the salons sampled. Results from the analysis of the questionnaires revealed that the effects of exposure to formaldehyde were experienced by almost all workers. The results of the study also revealed that hairdressers in salons that provide the entire range of salon services captured in the study are at higher risk of being exposed to the effects of formaldehyde. It is recommended that appropriate governmental agencies should take necessary measures to check and regulate the formaldehyde content in cosmetic products and condition under which they are used in the country.

## Figures and Tables

**Table 1:** Levels of formaldehyde and hazard quotients of the various salons in the submetros.

	*n*	Maximum (*µ*g/m^3^)	Minimum (*µ*g/m^3^)	Mean (*µ*g/m^3^)	Standard deviation (*µ*g/m^3^)
Bantama	6	434	33	170.67	152.41
Tafo	6	200	105	156.33	42.43
Kwadaso	6	349	24	153.50	110.84
Manhyia	6	166	102	126.17	23.47
Suame	6	166	21	88.67	50.46
Oforikrom	6	327	81	133.67	95.62
Asawase	6	268	21	114.33	91.64
Subin	6	265	24	136.67	88.47
Asokwa	6	157	97	125.17	25.62
Nhyiaso	6	187	35	170.67	152.41

**Table 2:** Statistical analysis of independent variables.

Services rendered	Type of service	Frequency	Percent	Valid percent	Cumulative percent
	Hair	60	—	—	—
	Nail	46	—	—	—
	Skin	1	—	—	—
	Eyelash/eyebrow	7	—	—	—
	Makeups	15	—	—	—

Number of services	One service	13	21.7	21.7	21.7
	Two services	32	53.3	53.3	75.0
	Three services	8	13.3	13.3	88.3
	Four services	6	10.0	10.0	98.3
	Five services	1	1.7	1.7	100
	Total	60	100.0	100.0	—

Average number of customers in a week	Less than 20	1	1.7	1.7	1.7
	20–29	28	46.7	46.7	48.3
	30–39	24	40.0	40.0	88.3
	40–49	6	10.0	10.0	98.3
	50 and above	1	1.7	1.7	100
	Total	60	100	100	—

Age of facility	Less or equal to 5 years	47	78.3	78.3	78.3
	More than five years	13	21.7	21.7	100
	Total	60	100	100	—

Years of working experience	0–5 years	29	48.3	48.3	48.3
	6–10 years	26	43.3	43.3	91.7
	11–15 years	4	6.7	6.7	98.3
	16 years and above	1	1.7	1.7	100
	Total	60	100	100	—

Academic qualification of owners/workers	JHS	50	83.3	83.3	83.3
	SHS	3	5.0	5.0	88.3
	NVTI	6	10.0	10.0	98.3
	None	1	1.7	1.7	100
	Total	60	100	100	—

Knowledge about chemical composition of products used	Yes	1	0.67	0.67	0.67
	No	59	99.33	99.33	100
	Total	60	100	100	—

Health effects	Respiratory irritation	37	—	—	—
	Eye irritation	27	—	—	—
	Nose irritation	20	—	—	—
	Skin irritation	16	—	—	—
	Nausea	—	—	—	—
	Vomiting	—	—	—	—
	Asthma	—	—	—	—
	Headache	4	—	—	—

**Table 3:** Correlation between dependent variables and independent variables.

	Formaldehyde concentration (*μ*g/m^3^)	Temperature (°C)	Academic qualification	Years of working experience	Age of facility	Number of services	Average number of customers in a week
Formaldehyde concentration (*μ*g/m^3^)	1	—	—	—	—	—	—
Temperature (°C)	0.307^*∗∗*^	1	—	—	—	—	—
Academic qualification	0.087	0.156	1	—	—	—	—
Years of working experience	0.314^*∗∗*^	0.229^*∗*^	0.075	1	—	—	—
Age of facility	0.418^*∗∗*^	0.082	−0.062	0.353^*∗∗*^	1	—	—
Number of services	0.315^*∗∗*^	0.180	0.383^*∗∗*^	−0.004	0.036	1	—
Average number of customers in a week	0.545^*∗∗*^	0.231^*∗*^	0.289^*∗*^	0.439^*∗∗*^	0.203	0.206	1

^*∗∗*^Correlation is significant at the 0.01 level (1-tailed). ^*∗*^Correlation is significant at the 0.05 level (1-tailed).

**Table 4:** Multiple linear regression model summary and overall fit statistics.

Mode	*R*	*R* square	Adjusted *R* square	Std. error of the estimate
1	0.690^a^	0.476	0.417	61.916
2	0.690^b^	0.476	0.428	61.348
3	0.679^c^	0.461	0.422	61.650
4	0.663^d^	0.440	0.410	62.306

^a^Predictors: average number of customers in a week, age of facility (beauty salon), number of services, temperature (°C), academic qualification, and years of working experience. ^b^Predictors: average number of customers in a week, age of facility (beauty salon), number of services, temperature (°C), and academic qualification. ^c^Predictors: average number of customers in a week, age of facility (beauty salon), number of services, and temperature (°C). ^d^Predictors: average number of customers in a week, age of facility (beauty salon), and number of services. ^e^Dependent variable: formaldehyde concentration (CL (*μ*g/m^3^)).

**Table 5:** ANOVA.

Model		Sum of squares	d*f*	Mean square	*F*	Sig.
1	Regression residual total	184931.762	6	30821.960	8.040	0.000^a^
		203179.171	53	3833.569		
		388110.933	59	—		
2	Regression residual total	184880.7906	5	36976.181	9.825	0.000^b^
		203230.1027	54	3763.519		
		388110.933	59	—		
3	Regression residual total	179069.936	4	44767.484	11.779	0.000^c^
		209040.998	55	3800.745		
		388110.933	59	—		
4	Regression residual total	170715.448	3	56905.149	14.658	0.000^d^
		217395.486	56	3882.062		
		388110933	59	—		

^a^Predictors: average number of customers in a week, age of facility (beauty salon), number of services, temperature (°C), academic qualification, and years of working experience. ^b^Predictors: average number of customers in a week, age of facility (beauty salon), number of services, temperature (°), and academic qualification. ^c^Predictors: average number of customers in a week, age of facility (beauty salon), number of services, and temperature (°C). ^d^Predictors: average number of customers in a week, age of facility (beauty salon), and number of services. ^e^Dependent variable: formaldehyde concentration (CL (*μ*g/m^3^)).

**Table 6:** Multiple linear regression estimates including the intercept and the significance levels.

Model		Unstandardized coefficients		Standardized coefficients	*T*	Sig.
		*B*	Std. error	Beta	
1	Constant	−268.119	146.782	—	−1.827	0.073
	Temperature (°C)	7.267	4.708	0.162	1.543	0.129
	Academic qualification	−12.732	10.348	−0.138	−1.230	0.224
	Years of working experience	−1.597	13.870	−0.014	−0.115	0.909
	Age of facility (beauty salon)	59.091	20.969	0.303	2.818	0.007
	Number of services	20.397	9.465	0.137	2.155	0.036
	Average number of customers in a week	47.445	12.556	0.444	3.779	0.000
2	Constant	−266.598	144.844	—	−1.841	0.071
	Temperature (°C)	7.180	4.606	0.160	1.559	0.125
	Academic qualification	−12.741	10.253	−0.138	−1.243	0.219
	Age of facility (beauty salon)	58.372	19.833	0.299	2.943	0.005
	Number of services	20.537	9.301	0.238	2.308	0.032
	Average number of customers in a week	46.901	11.526	0.439	4.069	0.000
3	Constant	−256.037	145.308	—	−1.762	0.084
	Temperature (°C)	6.851	4.621	0.152	1.483	0.144
	Age of facility (beauty salon),	61.753	19.743	0.316	3.128	0.003
	Number of services	16.624	8.794	0.193	1.890	0.064
	Average number of customers in a week	43.451	11.242	0.406	3.865	0.000
4	Constant	−45.825	32.140	—	−1.426	0.159
	Age of facility (beauty salon)	62.855	19.939	0.322	3.152	0.003
	Number of services	18.443	8.801	0.214	2.096	0.041
	Average number of customers in a week	46.625	11.154	0.436	4.180	0.000

^a^Dependent variable: formaldehyde concentration (CL (*μ*g/m^3^)).

**Table 7:** Excluded variables.

Model		Beta	*t*	Sig.	Partial correlation	Collinearity statistics
						Tolerance
2	Years of working experience	−0.014^a^	−0.115	0.909	−0.016	0.707
3	Years of working experience	−0.015^b^	−0.124	0.902	−0.017	0.707
	Academic qualification	−0.138^b^	−1.243	0.219	−0.167	0.790
4	Years of working experience	0.013^c^	0.112	0.912	0.015	0.726
	Academic qualification	−0.128^c^	−1.140	0.259	−0.152	0.792
	Temperature (°C)	0.152^c^	1.483	0.144	0.196	0.927

^a^Predictors in the model: constant, average number of customers in a week, age of facility (beauty salon), number of services, temperature (°C), and academic qualification. ^b^Predictors in the model: constant, average number of customers in a week, age of facility (beauty salon), number of services, and temperature (°C). ^c^Predictors in the model: constant, average number of customers in a week, age of facility (beauty salon), and number of services. ^d^Dependent variable: formaldehyde concentration (CL (*μ*g/m^3^)).

## Data Availability

The data used to support the findings of this study are included within the article.

## Supplementary Materials

Supplementary Materials Table S1: calculated hazard quotients for the salons under study.
