# Supplementary material for: Formaldehyde Exposure and Its Potential Health Risk in Some Beauty Salons in Kumasi Metropolis
Source: J Toxicol. 2020 Nov 5;2020:8875167. doi: 10.1155/2020/8875167 (PMC7661109; doi:10.1155/2020/8875167)
Supplement: Supplementary Materials — Table S1: calculated hazard quotients for the salons under study. [file 8875167.f1.docx]

**Supplementary Material**

**Table S1.** Calculated Hazard Quotients for the salons under study

| No. | Name of Salon | HCHO Conc. (μg/m^3^) | Hazard Quotient (HQ) |
| --- | --- | --- | --- |
| 1 | Sandy Hair care | 434 | 4.2 |
| 2 | Salina Hair care | 257 | 2.5 |
| 3 | Blessing Beauty Salon | 152 | 1.5 |
| 4 | Nyarko Beauty Salon | 48 | 0.5 |
| 5 | Grace Beauty Salon | 100 | 1.0 |
| 6 | Kate Beauty Salon | 33 | 0.3 |
| 7 | Gisela Beauty Salon | 152 | 1.5 |
| 8 | Vernadu Beauty Salon | 195 | 1.9 |
| 9 | Portapp Beauty Salon | 105 | 1.0 |
| 10 | Amet Beauty Salon | 200 | 1.9 |
| 11 | Annette Beauty Salon | 107 | 1.0 |
| 12 | Classic Hair Beauty Salon | 179 | 1.7 |
| 13 | Psalm 91 Beauty Salon | 101 | 1.0 |
| 14 | Thy Grace Beauty Salon | 197 | 1.9 |
| 15 | Gratefulness Beauty Salon | 24 | 3.4 |
| 16 | Joyce Beauty Salon | 349 | 1.3 |
| 17 | Eye Adom Beauty Salon | 134 | 1.1 |
| 18 | Vision Parlour Beauty Salon | 116 | 1.3 |
| 19 | Queen’s Salon | 133 | 1.3 |
| 20 | Fausty’s Salon | 106 | 1.0 |
| 21 | Vera’s Beauty Salon | 133 | 1.3 |
| 22 | Unique Lady’s Beauty Salon | 102 | 1.0 |
| 23 | Liberty Beauty Salon | 117 | 1.1 |
| 24 | Maa Tess Beauty Salon | 166 | 1.6 |
| 25 | Doris Beauty Salon | 74 | 0.7 |
| 26 | God’s Time is the Best Salon | 98 | 0.9 |
| 27 | Obaa Yaa Palace | 166 | 1.6 |
| 28 | Stella Salon | 56 | 0.5 |
| 29 | Sie Sie me Beauty Salon | 21 | 0.2 |
| 30 | Adonai Salon | 117 | 1.1 |
| 31 | Eyelashes Beauty Salon | 97 | 0.9 |
| 32 | Victoria’s Salon | 81 | 0.8 |
| 33 | Classic Lady Beauty Salon | 92 | 0.9 |
| 34 | All Shall Pass Beauty Salon | 327 | 3.2 |
| 35 | Lasty Vee Beauty Salon | 86 | 0.8 |
| 36 | Exodus 14;14 Beauty Salon | 119 | 1.2 |
| 37 | Mercy’s Beauty Salon | 85 | 0.8 |
| 38 | Mary’s Beauty Salon | 90 | 0.9 |
| 39 | Unique Beauty Salon | 268 | 2.6 |
| 40 | Aisha Hair Do | 175 | 1.7 |
| 41 | Maria Beauty Salon | 21 | 0.2 |
| 42 | Mavis Beauty Salon | 47 | 0.5 |
| 43 | Nhyira Beauty Salon | 193 | 1.9 |
| 44 | Aseda Nka Boafo Salon | 72 | 0.7 |
| 45 | Gina Hair and Nail Salon | 265 | 2.6 |
| 46 | Papaye Beauty Salon | 24 | 0.2 |
| 47 | Ange’s Beauty Salon | 97 | 0.9 |
| 48 | Thy Faithfulness Beauty Salon | 169 | 1.6 |
| 49 | Euniel’s Salon | 104 | 1.0 |
| 50 | Care Care Beauty Salon | 97 | 0.9 |
| 51 | Lizzy’s Beauty Salon | 132 | 1.3 |
| 52 | The Beauty Studio | 157 | 1.5 |
| 53 | Enye Mahoden Salon | 109 | 1.1 |
| 54 | Blessing Beauty Salon | 152 | 1.5 |
| 55 | Jane Beauty Salon | 35 | 0.3 |
| 56 | Aseda Beauty Salon | 78 | 0.8 |
| 57 | Gina’s Salon | 67 | 0.7 |
| 58 | Mathilda’s Beauty Salon | 187 | 1.8 |
| 59 | Adom Beauty Salon | 58 | 0.6 |
| 60 | Lady Nash Beauty Salon | 176 | 1.7 |
